# Unveiling the mechanism of dodecylphosphorylcholine as an extremely promising drug delivery system: From self-assembly clusters to drug encapsulation pathways

**DOI:** 10.1371/journal.pone.0320737

**Published:** 2025-05-07

**Authors:** Qijiang Shu, Linjing Yang, Li Li, Zedong Lin, Pengru Huang

**Affiliations:** 1 Institute of Information, Yunnan University of Chinese Medicine, Kunming, Yunnan, China; 2 Yunnan Key Laboratory of Dai and Yi Medicines, Yunnan University of Chinese Medicine, Kunming, Yunnan, China; 3 Yunnan Traditional Chinese Medicine Prevention and Treatment Engineering Research Center, Yunnan University of Chinese Medicine, Kunming, Yunnan, China; 4 Department of Science and Technology, Yunnan University of Chinese Medicine, Kunming, Yunnan, China; 5 School of Materials Science and Engineering, Taizhou University, Taizhou, Zhejiang, China; 6 Guangdong Provincial Key Lab of Nano-Micro Materials Research, School of Advanced Materials, Shenzhen Graduate School, Peking University, Shenzhen, Guangdong, China; 7 Guangxi Key Laboratory of Information Materials and Guangxi Collaborative Innovation Center of Structure and Property for New Energy and Materials, School of Material Science & Engineering, Guilin University of Electronic Technology, Guilin, Guangxi, China; University of Waterloo, CANADA

## Abstract

Significant progress has been achieved in cancer treatment with Doxorubicin (DOX), yet its low toxicity and poor bioavailability have long troubled scientists. Dodecylphosphorylcholine (DPC), as a candidate material for drug delivery systems (DDS), holds promise in assisting DOX to overcome its application bottleneck. In this study, employing a combination of quantum chemical calculations and molecular simulations, we delve into the dynamic processes of the interaction between DPC and DOX molecules for the first time. The results indicate that, under the synergistic effect where electrostatic repulsion plays a minor role and van der Waals attraction predominates, the end (containing choline group) of DPC molecules aggregate, self-assembling into multiple molecular clusters. There is a notable presence of electrostatic attraction and van der Waals attraction between DPC and DOX, which drives the adsorption or encapsulation of DOX molecules by DPC molecular clusters, thus presenting a favorable drug-loading conformation. During these processes, a substantial number of DPC molecules aggregate around DOX, with typical distances for interaction around 0.5 nm. The shape and position of DPC-DOX molecular clusters undergo significant dynamic changes within a simulated time of 0–70 ns, stabilizing thereafter. Our findings elucidate the interaction mechanism between DPC and DOX at the molecular scale, paving new avenues for the experimental synthesis of promising DDS eagerly sought by DOX.

## Introduction

Cancer remains one of the leading causes of mortality worldwide [1]. In 2023, the global number of cancer-related deaths reached 9.6 million, posing a significant challenge to public health. Despite continuous advancements in anticancer therapeutics, their clinical applications still face multiple challenges, such as low bioavailability, increased drug resistance, and severe toxic side effects [2]. Doxorubicin (DOX), a broad-spectrum antitumor drug, has demonstrated significant efficacy in the treatment of various cancers, including breast cancer, lung cancer, and lymphoma [3–5]. However, its clinical application is limited by severe cardiotoxicity and poor water solubility [6]. To overcome these limitations, researchers have been dedicated to developing efficient drug delivery systems (DDS) to enhance DOX targeting, reduce toxic side effects, and optimize its pharmacokinetics and biodistribution in vivo [7–11].

Currently, various DDS, including liposomes, polymeric nanoparticles, and protein-based nanocarriers, have been extensively studied and have shown improvements in the pharmacokinetics of DOX to some extent [12–14]. Among them, liposomes are one of the most established DDS, widely used for DOX delivery due to their excellent biocompatibility and high drug-loading capacity [12]. However, liposomal DDS still suffer from several limitations, such as inadequate stability, difficulty in precisely controlling drug release, and complex fabrication processes. Moreover, certain liposomes may trigger immune responses in vivo, leading to accelerated clearance and reduced drug delivery efficiency [15,16]. Therefore, the exploration of novel DDS materials remains a crucial research direction.

Dodecylphosphorylcholine (DPC), a phospholipid-based surfactant, demonstrates potential advantages in biocompatibility, structural stability, and drug-loading capacity. Firstly, compared to traditional polymers, DPC, due to its high similarity to biological membrane components, effectively reduces immunogenicity and toxic side effects, while prolonging the drug’s circulation time in vivo [17]. Secondly, in contrast to liposomes and protein-based nanocarriers, DPC can spontaneously form stable molecular clusters without the need for complex preparation processes, maintaining high structural stability under appropriate conditions and reducing the risk of drug inactivation [18,19]. Unlike liposomes, which are primarily used for hydrophobic drug delivery, DPC possesses both hydrophilic and hydrophobic properties, making it suitable for a wider range of drug types and enabling functional modifications to enhance targeting specificity and transmembrane capability [18]. Moreover, DPC is expected to form stable complexes with DOX through electrostatic interactions and van der Waals forces, which could enhance drug-loading efficiency and enable more controlled drug release [20].

Despite the numerous potential advantages of DPC in DDS, research on its application for DOX loading remains largely unexplored. In this study, a combination of quantum chemical calculations and molecular simulations is employed to systematically investigate the self-assembly of DPC into molecular clusters and its interaction mechanism with DOX. Special emphasis is placed on analyzing the stability of the DPC-DOX complex and its drug encapsulation properties. By elucidating the mechanistic role of DPC as a DDS, this study aims to provide a theoretical foundation for novel DOX delivery strategies and offer new insights for the future optimization of DDS design.

## Materials and methods

### Conformational search of DOX and DPC molecules

Coordinates of the three-dimensional structures of DOX and DPC are obtained from the PubChem website [21]. Utilizing the Sobtop software [22], top files for DOX and DPC molecules are constructed based on the GAFF force field. Subsequently, annealing simulations are conducted separately for DOX and DPC molecules. This process begins with energy minimization simulations. Following this, within each 200 ps time interval, the temperature is gradually increased from 0 K to 500 K, then smoothly decreased back to 0 K, saving coordinates periodically. This simulation continues for 10000 ps, generating 50 initial structures for each DOX and DPC molecule. The parameters in the Molclus software [23] are set to initiate the conformational search. First, the Mopac software performs geometry optimization using the semi-empirical quantum chemistry method PM7 [24], based on the tight-binding theory. For the optimized structures, conformers with a free energy difference of less than 0.25 kcal/mol are considered identical, and redundant structures are removed to improve computational efficiency. Subsequently, the optimized conformers undergo further refinement using the Orca software [25] through a two-step density functional theory optimization process. Initially, the B97-3c functional is applied in combination with the def2-mTZVP basis set [26]. This is followed by a more precise optimization using the PWPB95 functional [27] with D3 dispersion correction [28] and the def2-QZVPP basis set [29,30]. Finally, the optimized conformers are ranked based on their energy, and the most stable conformations of DOX and DPC molecules are selected as the foundation for subsequent simulations.

### Molecular dynamics simulation of the system

Utilizing the Multiwfn software [31], the RESP2 (0.5) charges [32] of the stable conformations of DOX and DPC are calculated. Subsequently, their respective top files are generated using the Sobtop software. A cubic simulation box of dimensions 10 × 10 × 10 nm^3^ is constructed, wherein 3 DOX molecules and 150 DPC molecules are randomly inserted. The solvent model employed is PIP4P, and the simulation box (the simulation system) undergoes solvation treatment.

Energy minimization simulations are performed on the system using the steepest descent method [33], with a convergence criterion set at 1000 kJ/mol/nm, a time step of 2 fs, and a maximum of 50000 steps. Subsequently, the system is coupled to 300 K using the V-rescale algorithm to achieve the NVT ensemble, with a simulation time of 1000 ps. Then, the Berendsen algorithm is employed to control the system pressure to stabilize at 1.0 bar, obtaining the NPT ensemble, with a simulation time of 1000 ps. Finally, a 100 ns-long simulation is conducted to obtain the final product. NVT, NPT, and 100 ns simulations all employ the leap-frog integration algorithm with a time step of 2 fs. The LINCS algorithm is used to constrain bonds involving hydrogen atoms. The Particle Mesh Ewald method is employed to compute electrostatic interactions, with a cutoff radius of 1.2 nm for electrostatic and Lennard-Jones interactions [33].

### Computational and analytical methods

To observe molecular structures and generate visualizations, ChemiQ, Gaussianview [34], and VMD software [35] are utilized. The molecular surface electrostatic potentials of DOX and DPC molecules are computed using Multiwfn software. Additionally, colored molecular surface electrostatic potential distributions are plotted by combining Multiwfn and VMD software. The xTB software, using the semi-empirical quantum calculation method GFN-xTB2 [36] based on an improved generalized tight-binding model, is employed to perform single-point energy calculations on the selected part of the simulated system of interest. The acquired structural and wavefunction data is imported into the Multiwfn software to calculate the difference function of the molecular electron density gradient (δg). Subsequently, utilizing the IGMH (Independent Gradient Model based on Hirshfeld partition) method [37], this information is integrated with VMD software to visualize the interaction regions between the components.

All molecular simulation systems are subjected to periodic boundary conditions in three dimensions, and the simulations are performed using the GROMACS software [33]. Based on the trajectory files obtained from the simulations, the ‘gmx trjconv’ command is executed to correct for artifacts introduced by periodic boundary conditions. The ‘gmx rms’ command is employed to extract the root mean square deviation (RMSD) of the computed groups. The ‘gmx gyrate’ command is utilized to extract the time evolution data of the gyration radius of the computed groups. The ‘gmx mindist’ command is run to extract the number of atomic contacts between the groups of interest. The ‘gmx rdf’ command is employed to compute and extract the radial distribution function (RDF) between the groups of interest. The ‘gmx hbond’ command is used to quantify the number of hydrogen bonds between components of interest in the simulated system. Lastly, the ‘gmx energy’ command is executed to compute and extract the interaction energies (electrostatic and van der Waals energies) between components of interest in the system.

## Results and discussion

### Conformational analysis of DOX and DPC molecules

**Fig 1:**
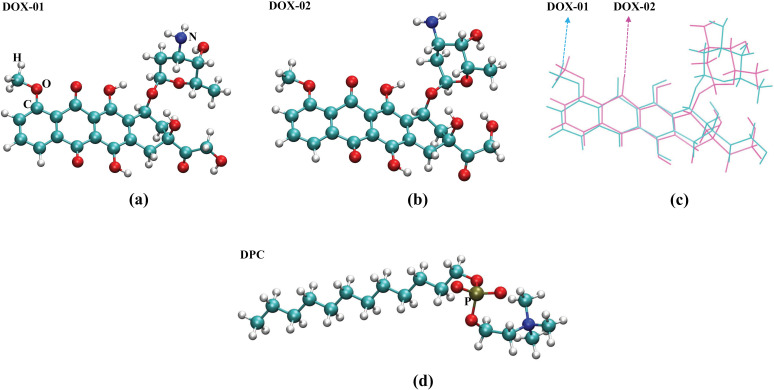
(a) and (b): Two relatively most stable conformations of the DOX molecule. (c): Result of overlaying the two structures from (a) and (b) to illustrate the differences between structures. The two structures are depicted in different colors for clarity. (d): The relatively most stable conformation of the DPC molecule.

DOX and DPC are flexible molecules with numerous rotatable bonds within their structures, leading to multiple local minima on their potential energy surfaces. As described in the Materials and methods section, we employ a molecular dynamics simulation approach involving simulated annealing, wherein DOX and DPC molecules traverse energy barriers and transition to other potential well regions at elevated temperatures, subsequently becoming trapped near local minima as the temperature decreases. Multiple rounds of annealing simulations are conducted, with each simulation saving a structure. These structures, distributed at different positions on the potential energy surface, are used as initial guess structures. Subsequent geometric optimizations and energy rankings are performed on these structures individually to identify the most stable conformations of the molecules. The corresponding results are depicted in Fig 1 and Table 1.

**Table 1:** Parameter differences between two relatively most stable conformations of the DOX molecule, in which the RMSD value is calculated by overlaying the two structures with one of them as the reference (DOX-1 as reference, RMSD set to 0).

Conformation	Free energy (a.u.)	RMSD (nm)	Boltzmann distribution ratio at 300 K (%)
DOX-01	-1928.247646	0.000000	99.35
DOX-02	-1928.242861	1.326480	0.65

Figs 1(a) and (b) depict two low-energy conformations of DOX obtained from the search. For ease of comparing the structural differences between the two conformations, (c) shows the result of overlaying them. Quantitatively, Table 1 provides the root mean square deviation (RMSD) [38] calculated from overlaying the two conformations with conformation 1 (DOX-01) as the reference. Table 1 also displays the free energies of the two conformations and their Boltzmann distribution ratios at 300 K, where the distribution ratio of DOX-01 is as high as 99.35%. Fig 1(d) presents the lowest-energy conformation of DPC obtained through conformational searching. Within the predefined search range (detailed in the Materials and methods section), the free energy differences between this conformation and other identified conformations remain below the specified threshold, leading to their classification as equivalent. Based on these results, the conformations depicted in Figs 1(a) and (d) are selected for constructing the simulation system to conduct subsequent molecular dynamics studies.

### Configuration evolution of the simulated system

**Fig 2:**
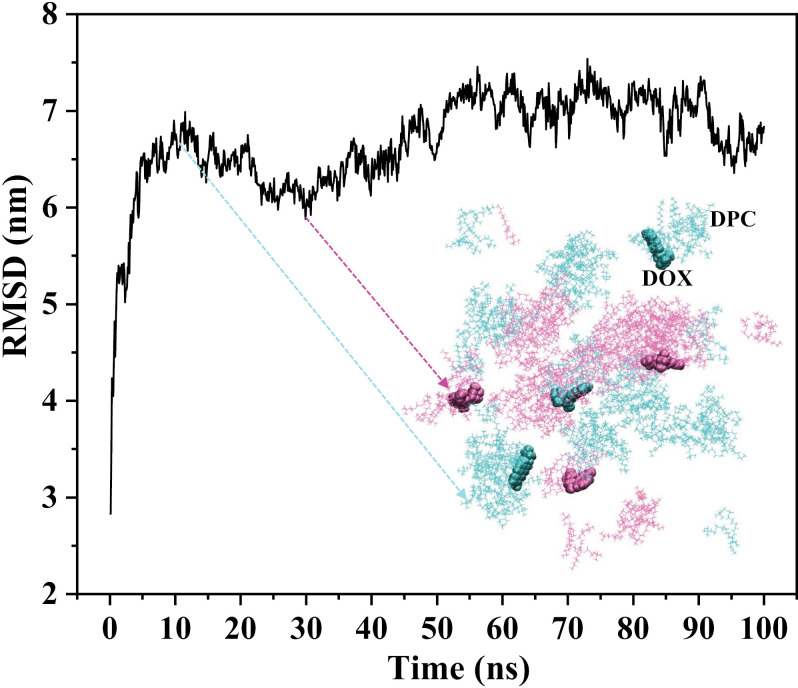
RMSD variation of the DPC-DOX molecular system over simulation time. The inset depicted in green represents the overall configuration of the molecular system at 12 ns, where DPC molecules are illustrated in branch-like shapes and DOX molecules are represented as spheres. The inset in red corresponds to the overall configuration of the molecular system at 31 ns. Both system configurations at these time points are overlaid to facilitate the visualization of their differences.

Three DOX molecules and 150 DPC molecules are randomly dispersed within a water-filled cubic box with a side length of 10 nm. A simulation of 100 ns duration is conducted on this system (see the Materials and Methods section for simulation details). The temporal variation of the RMSD of the entire DOX-DPC system is depicted in Fig 2. The RMSD values exhibit rapid increments within the time interval of 0–15 ns, indicating substantial alterations in the overall structural arrangement of the system. Subsequently, from 15 to 100 ns, the RMSD remains within a certain range, a fluctuation easily comprehended as the DOX-DPC system at this juncture comprises multiple molecular clusters, whose shapes and positions evolve over time. Configurations of the system at 12 ns and 31 ns are overlaid and represented in Fig 2 with distinct colors, serving as aids in comprehending the dynamic transformations of molecular clusters. Evidently, the qualitative changes in RMSD values reflect the extent of variations in the overall structural configuration of the system. The minor fluctuations in RMSD values during the final 30 ns (70–100 ns) timeframe suggest that the system’s overall structural arrangement has attained stability.

The number of atomic contacts between molecules can describe the structural changes of a system composed of multiple molecules. Here, the number of atomic contacts between molecules is calculated using the following formula:


$$N_{C}(t)=\sum_{i=1}^{N_{A}}\sum_{j=1}^{N_{B}}\int_{r_{i}}^{r_{i}+0.6\;nm}\delta \left(r(t)-r_{j}(t)\right)dr$$ (1)

**Fig 3:**
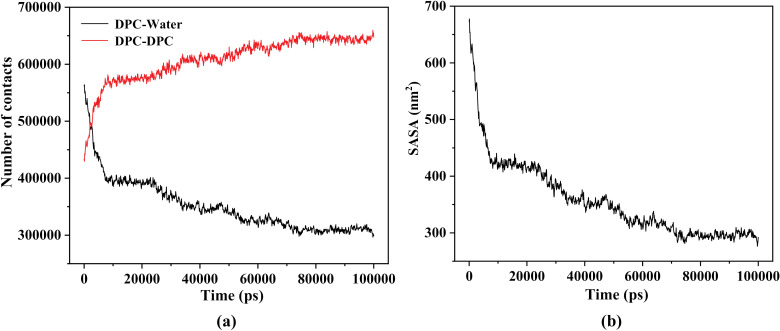
(a): The temporal evolution of the contact numbers between DPC molecules and water molecules (DPC-Water), as well as between DPC molecules themselves (DPC-DPC). (b): Changes in the solvent accessible surface area (SASA) of the DPC molecular system over simulation time.

Where $N_{A}$ and $N_{B}$ represent the total number of atoms in molecules A and B, respectively, and $r_{i}\;$ is the distance from the *j*-th atom in molecule B to the *i*-th atom in molecule A. Fig 3(a) depicts the atomic contact numbers between all DPC and water molecules (DPC-Water) as well as between all DPC molecules (DPC-DPC) in the simulated system. As shown in Fig 3(a), during the initial approximately 15 ns of simulation, there is a rapid decrease in DPC-Water contacts and a rapid increase in DPC-DPC contacts, suggesting a significant aggregation of DPC molecules originally dispersed in water. The curve variations observed between 15 and 70 ns indicate a gradual slowdown in the molecular clustering process. During the 70–100 ns timeframe, the curves stabilize at a constant value, indicating that the molecular clusters reach an equilibrium state, which is consistent with the conclusions drawn from the RMSD analysis in Fig 2. Another piece of evidence supporting this inference is the calculation of the solvent accessible surface area (SASA), which is obtained using the same methods as reported in some previous studies [39,40]. Fig 3(b) illustrates the SASA of the entire DPC molecular system. Similar to the trend observed in DPC-Water contacts, the SASA values rapidly decrease after the initiation of the simulation and maintain a low level during the final time period. The decrease in SASA serves as compelling evidence for the transition of molecules from dispersion to aggregation, while its stability in the later stage corresponds to the formation of stable molecular clusters. The aggregation of surfactant molecules in solution inhibits their contact with water molecules, and this unfavorable interaction between hydrophobic groups and water is one of the potent forces driving the self-assembly of polymer micelles in aqueous solutions. The numerical values presented in Fig 3 reflect the changes in this unfavorable interaction during the process of molecular aggregation.

**Fig 4:**
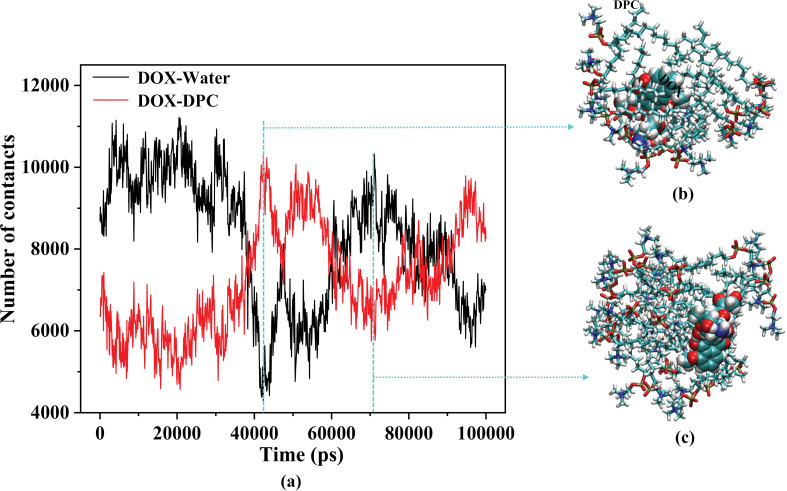
(a): The variation of the contact numbers between DOX molecules and water molecules (DOX-Water), as well as between DOX molecules and DPC molecules (DOX-DPC), over simulation time. (b) and (c): Representative molecular clusters extracted from the simulation system at 43 ns and 71 ns, respectively, where DPC are depicted by colored sticks and DOX are represented by colored spheres, vividly illustrating the contact situation between DOX and DPC.

During the process of DPC molecules aggregating to form clusters, a focal point of interest is whether the DOX drug molecules in the system interact with the clusters as expected. This can be analyzed through changes in their relative positional relationships. In Fig 4(a), the overall trend of the curves shows a decrease in the contact number between DOX and water molecules (DOX-Water) and an increase in the contact number between DOX and DPC molecules (DOX-DPC). This corresponds to the transition of DOX molecules from being dispersed in water to being adsorbed (or encapsulated) by DPC clusters. Upon closer examination, significant fluctuations in the intermolecular contact numbers are observed within the time range of 40–100 ns as depicted in Fig 4(a). Fig 4(b) and (c) respectively depict local snapshots extracted from the simulation system at 43 ns and 71 ns. In Fig 4(b), DOX is buried within the DPC clusters, resulting in fewer DOX-Water contacts and more DOX-DPC contacts. Conversely, in Fig 4(c), the position of DOX shifts to the exterior of the DPC clusters, leading to an increase in DOX-Water contacts and a decrease in DOX-DPC contacts. With only three DOX molecules in the system, the counted contact numbers are highly sensitive to the positional changes of these three DOX molecules, which explains the significant fluctuations in the numerical values of the curves in the figure.

The distribution of DPC molecules and water molecules around DOX can be further analyzed using the radial distribution function (RDF), which is described by the following equation [41]:


$$g_{DOX-B}(r)=\frac{\left\langle \rho_{B}(r)\right\rangle }{{\left\langle \rho_{B}\right\rangle }_{local}}\frac{1}{{\left\langle \rho_{B}\right\rangle }_{local}}\frac{1}{N_{DOX}}\int_{i\in DOX}^{N_{DOX}}\int_{j\in B}^{N_{B}}\frac{\delta (r_{ij}-r)}{4\pi r^{2}}$$ (2)

**Fig 5:**
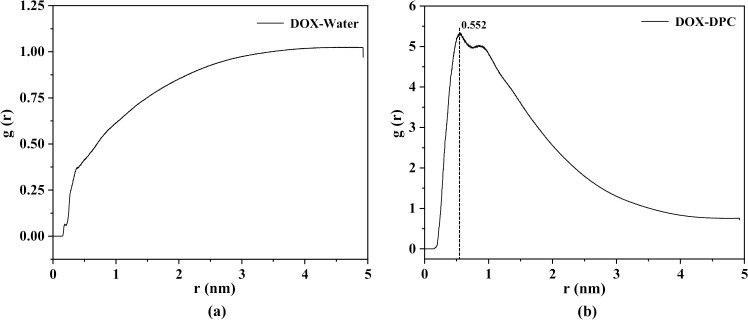
(a): Radial distribution function of water molecules around DOX. (b): Radial distribution function of DPC molecules around DOX.

Where $\left\langle \rho_{B}(r)\right\rangle$ represents the partial density of component B (DPC or Water) at a distance *r* from DOX, ${\left\langle \rho_{B}\right\rangle }_{local}$ is the partial density of component B in all spheres of radius *r* around DOX, $g_{DOX-B}(r)$ calculates the probability of finding DPC or Water in a *dr* shell at distance *r* from DOX as a reference point, and the results are displayed in Fig 5. As shown in the figure, at a shell position around 0.5 nm from DOX, the average value of DOX-Water is relatively small (Fig 5(a)), while DOX-DPC exhibits a high peak (Fig 5(b)), indicating that the space around DOX is predominantly occupied by DPC molecules, with water molecules being displaced. As *r* exceeds 3.0 nm, the average value of DOX-Water reaches its maximum and tends to stabilize, while DOX-DPC shows smaller values. This is consistent with the peripheral environment of molecular clusters being filled with water molecules and a few dispersed DPC molecules. The peak value in Fig 5(b) quantitatively indicates that the main distance for interaction between DOX and DPC molecules is approximately 0.552 nm.

**Fig 6:**
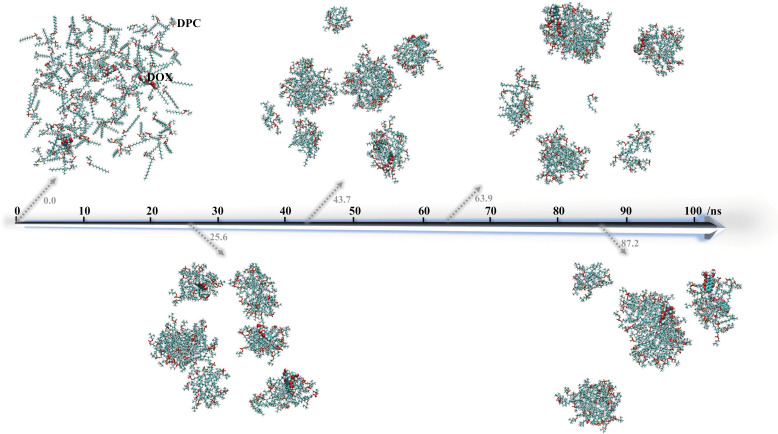
Snapshots of the system configuration at typical moments during the evolution of the DPC-DOX molecular system over simulation time. For clarity and comparison, DPC molecules are depicted using colored sticks, while DOX molecules are represented by colored spheres.

To visualize the evolution of the overall configuration of the simulated system over time, snapshots at random moments along the timeline are extracted and depicted in Fig 6. The moment at 0.0 ns corresponds to the initial state of the system, where three DOX molecules and 150 DPC molecules are randomly dispersed in the simulation box, with each molecule in an extended state as demonstrated in Fig 1. As the simulation progresses, at 25.6 ns, multiple molecular clusters form in the system, with DOX molecules being adsorbed or encapsulated within different clusters. At this point, the majority of DOX and DPC molecules are in a compact state, consistent with the fact that they have undergone a dynamics process dominated by interactions. At 43.7 ns, the shapes and sizes of molecular clusters change, including alterations in the relative positions between DOX and the clusters. At 63.9 ns and 87.2 ns, the molecular clusters in the system become significantly larger, leading to a decrease in the number of clusters. Although the shapes and positions of molecular clusters continue to fluctuate, the evolution characteristics of the concerned DPC-DOX configurations are considered to be stabilizing, corresponding to the stabilization trend observed in the RMSD values in Fig 2. Fig 6 effectively aids in understanding the self-assembly process of DPC molecules aggregating into multiple clusters at various evolving sites, crucially demonstrating that these DPC clusters can adsorb or encapsulate DOX to exhibit favorable drug-loading morphologies. Clearly, Fig 6 serves as the most intuitive verification, ensuring the credibility of the core conclusions speculated in Figs 3-5.

### Analysis of the interaction between DOX and DPC molecules

**Fig 7:**
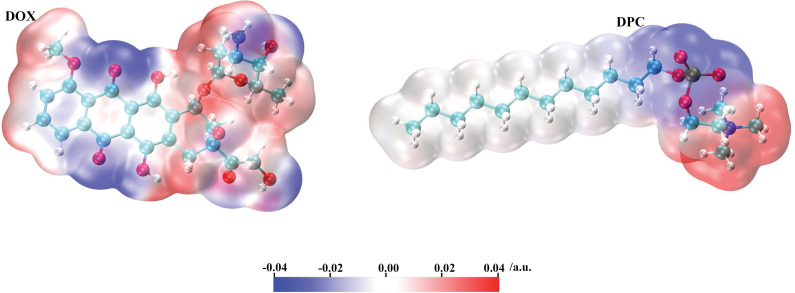
ESP mapped the van der Waals surface of DOX and DPC molecules. Significant surface local minima and maxima of the ESP are depicted in blue and red, respectively. The unit is in a.u..

Understanding the essence of the interaction between DOX and DPC is crucial for comprehending the formation mechanism of DOX-DPC clusters and thereby guiding experimental design for the development of key DPC DDS. In the early stages of interaction, molecules approach each other through electrostatic attraction. Positive regions on the molecular surface tend to interact with negative regions, with a stronger tendency observed when the positive values are larger and the negative values are greater. Therefore, the distribution of molecular surface electrostatic potentials can be utilized to predict or explain the relative orientations of molecules in complexes, binding strengths, receptor-ligand binding modes, molecular adsorption, and other related issues [42]. For ease of analysis, the calculated van der Waals surface electrostatic potentials of DOX and DPC molecules are visualized using colored isosurfaces of electron density to obtain easily distinguishable surface distributions, as depicted in Fig 7. As shown in the figure, the negative electrostatic potential regions (blue) and positive electrostatic potential regions (red) in DOX are relatively evenly distributed. From left to right, the tail region of DOX’s tricyclic structure exhibits weak positive electrostatic potential, the waist region shows pronounced negative electrostatic potential, and the head region consists of both local positive and negative electrostatic potential areas. The dodecyl groups of DPC appear nearly neutral (white), while the head groups of phosphoryl and choline exhibit distinct surface negative and positive electrostatic potentials, respectively. This implies that, at the beginning of the simulation, DPC molecules predominantly aggregate by approaching each other or approaching DOX through their molecular headgroups (the choline end). Subsequently, they continuously adjust their positions to minimize the energy of the molecular clusters as the relative positions between molecules change.

**Fig 8:**
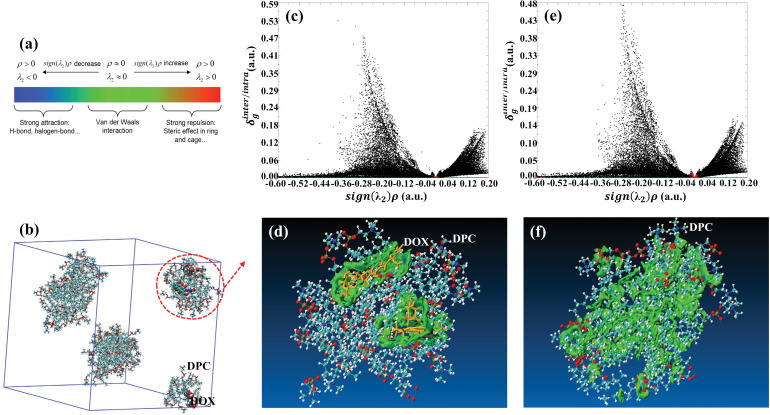
(a): Correspondence between different values of the $sign(\lambda_{2}rho$ function and types of interactions. (b): Overall configuration of the molecular system under simulation at 90 ns. The portion enclosed by the red dashed line is extracted as the object of interest. (c): Within the molecular clusters encircled in (b), systems composed of DOX molecules and DPC molecules are defined as two segments. After computation, scatter plots of the difference function of electron density gradients between segments, $\delta_{g}^{inter}$ vs $sign(\lambda_{2}rho$, are plotted (in red), as well as scatter plots of the difference function of electron density gradients within segments, $\delta_{g}^{intra}$ vs $sign(\lambda_{2}rho$ (in black). (d): Based on the computational results from (c), isosurface plots of $\delta_{g}^{inter}$ between segments are generated to illustrate the types and regions of interactions between segments. For clarity, DOX is represented by golden sticks, and DPC is represented by colored spheres. (e): Each DPC molecule within the molecular clusters encircled in (b) is individually defined as a segment. Scatter plots of $\delta_{g}^{inter}$ vs $sign(\lambda_{2}rho$ (in red) and $\delta_{g}^{intra}$ vs $sign(\lambda_{2}rho$ (in black) are plotted. (f): Corresponding to the results obtained in (e), isosurface plots of $\delta_{g}^{inter}$ between segments are generated to display the interaction regions and types between segments.


Based on the atoms-in-molecules (AIM) theory, the $sign(\lambda_{2}rho$ function can be defined to distinguish the types and strengths of interactions within the system, where ρ represents the actual electron density of the system, and $\lambda_{2}$ denotes the second-largest eigenvalue of the electron density Hessian matrix. The numerical values of $sign(\lambda_{2}rho$ correspond to the types of interactions, as shown in Fig 8(a), with different interaction types distinguished by color, and the depth of color indicating the strength of the interaction [37]. Additionally, a function $\delta_{g}$, defined as the difference in electron density gradient, can be expressed as follows [37,43]:

$$\delta_{g(r)}=\left|\sum iabs\left[\nabla \rho_{i}(r)\right]\right|\left|\sum i\nabla \rho_{i}(r)\right|$$ (3)

Here, i denotes the atomic index, ∇ρ represents the electron density gradient vector, and abs(∇ρ) denotes the absolute value of each component of the ∇ρ vector. The physical significance of *δg* is that the larger the value of *δg*, the stronger the interaction between atoms in that region.

To reveal the essence of the interaction between DOX and DPC molecules, the region of interest is extracted from the simulated system at 90 ns. This region, outlined by the red dashed line in Fig 8(b), is singled out for investigation. It is divided into two segments: Segment one consists of DOX molecules, while segment two contains all DPC molecules. The $\delta_{g}$function of the interaction region between these two segments is labeled as $\delta_{g}^{inter}$. It is being calculated and plotted as $\delta_{g}^{inter}$ vs $sign(\lambda_{2}rho$ in a scatter plot, as indicated by the red scatter dots in Fig 8(c). Meanwhile, the $\delta_{g}$ function of the interaction region among atoms within segments is labeled as $\delta_{g}^{intra}$. It is plotted as black dots in Fig 8(c). In Fig 8(c), there is a peak formed by red dots near the position where $sign(\lambda_{2}rho$ approaches 0. According to Fig 8(a), this peak indicates the presence of van der Waals interactions between the two segments. Additionally, in regions where $sign(\lambda_{2}rho$ is significantly greater than 0, there are numerous black dots, indicating steric hindrance within the system. In regions where $sign(\lambda_{2}rho$ exhibits large negative values, there are extensive scattering dots, suggesting areas of high electron density. These regions can be considered as corresponding to chemical bond regions within the segments. By employing the IGMH (Independent Gradient Model based on Hirshfeld partition) method [37], the isosurfaces of $\delta_{g}^{inter}$ are generated. The $sign(\lambda_{2}rho$ function is then projected onto these surfaces using different colors (as indicated by the color scale in Fig 8(a)), enabling a clear examination of the interaction regions between segments and the types and strengths of interactions. The results are depicted in Fig 8(d). In Fig 8(d), interaction regions are predominantly shown in green, indicating that the interactions between DOX and DPC molecules are primarily governed by van der Waals forces, which is consistent with the inference from Fig 8(c). In a few isolated regions of green, a noticeable central blue is displayed, corresponding to a small number of hydrogen bonds.

Furthermore, each DPC molecule within the object of study is individually designated as a segment. The scatter plot of ${\;\delta }_{g}\;$ between these segments ($\delta_{g}^{inter}$vs $sign(\lambda_{2}rho$, in red) and the scatter plot of $\;\delta_{g}\;$ within segments ($\delta_{g}^{intra}$vs $sign(\lambda_{2}rho$, in black) are illustrated in Fig 8(e). Similarly, a conspicuous peak of red dots is observed only at the position where $sign(\lambda_{2}rho$ approaches 0, indicating that the clustering configuration between these DPC molecules is primarily upheld by van der Waals forces, while the numerous black dots correspond to steric hindrance and chemical bonds within molecules. Their isosurface plots of $\delta_{g}^{inter}$ based on the IGMH model are displayed in Fig 8(f). Clearly, the interaction regions are predominantly green, signifying that the main driving force for the aggregation of DPC molecules is the van der Waals interactions between them. The conclusions derived from Fig 8(e) and (f) exhibit excellent consistency.

**Fig 9:**
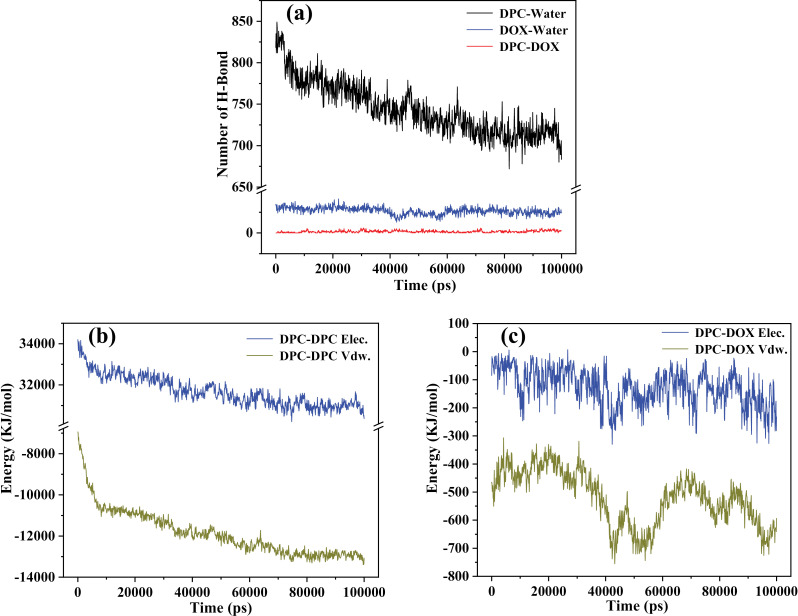
(a): The variation of hydrogen bond quantities between different molecules over simulation time in the simulated molecular system. (b): The change of electrostatic interaction energy (Elec.) and van der Waals interaction energy (Vdw.) between DPC molecules in the molecular system over simulation time. (c): The variation of Elec. and Vdw. between DPC molecules and DOX molecules in the molecular system over simulation time.

Typically, hydrogen bonds are characterized by a combination of cutoff values for the angle formed by the hydrogen donor-acceptor (30°) and the distance between the donor and acceptor (0.35 nm), where the hydrogen donor includes OH and NH groups, and the acceptor defaults to O and N [33]. Fig 9(a) illustrates the variation of hydrogen bond quantities over simulation time, as statistically derived from the simulation system. In the plot, the number of hydrogen bonds between DPC and water molecules (DPC-Water) noticeably decreases over simulation time. This reduction is attributed to the process wherein these DPC molecules transition from a state of random dispersion to clustered aggregation, resulting in a diminished contact with water molecules. Conversely, the number of hydrogen bonds between DOX and water (DOX-Water) does not exhibit a significant decrease over time. Similarly, the quantity of hydrogen bonds between DPC-DOX clusters also does not substantially increase despite their increased contact, with the DPC-DOX count remaining relatively small (in single digits). This suggests that hydrogen bonds do not play a significant role in the interactions between DOX and the surrounding water molecules and DPC molecules. This observation aligns with the absence of a large number of hydrogen bonds observed in Figs 8(c) and (d).

Fig 9(b) illustrates the variation of interaction energy between DPC molecules during the process of cluster formation over simulation time. The electrostatic interaction energy (Elec.) [44] between DPC molecules exhibits large positive values, indicating the presence of electrostatic repulsion between some DPC molecules, with their contribution to the system’s Elec. being higher than that of other DPC molecules involved in electrostatic attraction. The Elec. values slowly decrease during the initial 70 ns of simulation time and subsequently stabilize, suggesting that the distance between repulsive molecules in the system gradually increases, a process that becomes more pronounced compared to the process of attractive molecules reducing their separation distance due to electrostatic attraction. The van der Waals interaction energy (Vdw.) [45] between DPC molecules is negative, indicating a significant van der Waals attraction between them. Similarly, the Vdw. values notably decrease during the initial 70 ns of simulation time and then remain almost constant, corresponding to a process where mutually attractive molecules gradually shorten their intermolecular distances and eventually stabilize. Overall, the synergistic action of attraction and repulsion drives and maintains the system’s changes. Clearly, molecular aggregation guided by attraction predominates, as evidenced by the clustering of DPC molecules observed earlier. In addition, both the number of hydrogen bonds and the values of Elec. and Vdw. remain stable during the final ~ 30 ns of the simulation. This stability serves as direct evidence that intermolecular interactions no longer underwent significant fluctuations, which aligns well with the earlier conclusion that the system had reached a stable state.

Fig 9(c) displays the interaction energy between DPC and DOX. Both Elec. and Vdw. are negative values. As simulation time increases, all Elec. and Vdw. noticeably decrease, indicating that electrostatic attraction and van der Waals attraction are gradually bringing the DPC molecular system closer to DOX molecules, and the more significant decrease in Vdw. suggests that van der Waals forces predominantly drive this process. Specifically, van der Waals forces serve as the primary driving force for DPC clusters to adsorb DOX and form a stable drug delivery configuration, consistent with the observations in Fig 8(d). It is worth noting that the curves in Fig 9(c) exhibit relatively large fluctuations. This is due to the fact that the Elec. and Vdw. values for DPC-DOX are sampled from the very small number of DOX molecules in the system. Consequently, even slight positional variations of these few DOX molecules can significantly affect the statistical values. This observation is consistent with the discussion in Fig 4.

## Conclusions

We integrate various techniques such as quantum chemistry calculations and molecular dynamics simulations to obtain stable conformations of DOX and DPC. Based on this foundation, we construct a molecular simulation system consisting of 3 DOX and 150 DPC molecules randomly inserted into a water box, and the system undergoes a 100 ns dynamic process. The results indicate that DPC molecules aggregate at multiple sites to form several molecular clusters, which can adsorb or encapsulate DOX molecules, demonstrating favorable drug-loading configurations. The analysis of RMSD, intermolecular contact numbers, RDF, and other data reveals that the shapes, sizes, and relative positions of these molecular clusters with respect to DOX molecules are undergoing dynamic changes. This dynamic variation tends to stabilize around the last 30 ns of simulation time, with the typical distance between DPC and DOX interactions being approximately 0.5 nm. Furthermore, analysis of molecular surface electrostatic potentials, differences in electron density gradients ($\delta_{g}$), hydrogen bonds, and interaction energies indicates that DPC molecules tend to approach each other or move closer to DOX molecules using their phosphoryl and choline groups, thereby forming molecular clusters. The morphology of DPC molecular clusters is collectively driven and maintained by electrostatic repulsion and van der Waals attraction between DPC molecules, with van der Waals forces playing a predominant role. Significant electrostatic and van der Waals attractions exist between DPC and DOX molecules, serving as the primary driving forces for DPC molecules to adsorb and encapsulate DOX, forming stable drug delivery configurations. Remarkably, a significant quantity of hydrogen bonds is not observed during this process. Overall, we have gained molecular-level insights into the dynamic process of DPC self-assembly and its interaction with DOX. These results provide critical support for the feasibility of experimentally synthesizing DPC-based DDS and optimizing their preparation processes (self-assembly). Furthermore, they offer valuable guidance for strategies such as chemical modifications (phosphoryl and choline) and the regulation of intermolecular van der Waals interactions (e.g., adjusting hydrophobic chain length, solvent environment, and ionic strength) to fine-tune DPC’s affinity for drug molecules, self-assembly stability, and drug loading capacity. Our findings lay a theoretical foundation for the future experimental synthesis or design of potential DDS tailored for DOX.
